# Knowledge, Attitudes and Practices Regarding Taeniasis in Pakistan

**DOI:** 10.3390/diseases11030095

**Published:** 2023-07-07

**Authors:** Saba Bibi, Muhammad Kamran, Haroon Ahmad, Kainat Bibi, Syed Kamran Ul Hassan Naqvi, Qingqiu Zuo, Naseer Ali Shah, Jianping Cao

**Affiliations:** 1 Department of Biosciences, COMSATS University Islamabad (CUI), Park Road, Chak Shahzad, Islamabad 45550, Pakistan; 2 Department of Medical Laboratory Technology, Islamabad Medical and Dental College, Islamabad 45400, Pakistan; 3 National Institute of Parasitic Diseases, Chinese Center for Disease Control and Prevention (Chinese Center for Tropical Diseases Research), Shanghai 200025, China; 4 Key Laboratory of Parasite and Vector Biology, National Health Commission of the People’s Republic of China, Shanghai 200025, China; 5 WHO Collaborating Center for Tropical Diseases, Shanghai 200025, China; 6 The School of Global Health, Chinese Center for Tropical Diseases Research, Shanghai Jiao Tong University School of Medicine, Shanghai 200025, China

**Keywords:** taeniasis, *Taenia solium*/*saginata*, knowledge, attitudes, practices, Pakistan, socio-demographic

## Abstract

Taeniasis is a neglected zoonotic disease responsible for serious health disorders, such as seizures, and may even cause death. Humans are the definitive host for the three species *Taenia solium* (pork tapeworm), *T. saginata* (beef tapeworm), and *T. asiatica*, harboring the adult tapeworm in the small intestine. In this study, a structured questionnaire was circulated to assess the knowledge, attitudes, and practices (KAPs) regarding taeniasis among the rural and urban communities of Rawalpindi and Islamabad, Pakistan. A total of 770 individuals participated in the study. Of the total respondents, 44.4% had little knowledge about the disease and its impact, while the majority (70%) of respondents showed a willingness to participate in elimination campaigns by providing fecal samples. Most respondents kept raw meat separated from clean utensils (81.6%) and checked the internal temperature of meat when cooking it (75.1%). Regression analysis showed a significant association between age and knowledge, especially in the 20–30 years (*p* < 0.05; OR 0.574) and 30 to 40 years (*p* < 0.05; OR 0.553) age groups, and being a resident in Rawalpindi (*p* < 0.05; OR 0.68) and other cities (*p* < 0.05; OR 2.43), except Islamabad. Income ranges of 31,000–50,000 PKR (*p* < 0.05; OR: 0.574), 51,000–70,000 PKR (*p* < 0.05; OR 0.531), and above 70,000 PKR (*p* < 0.05; OR 0.42) were significantly related to attitude, compared with individuals with incomes of 10,000–30,000 PKR. Income above 70,000 PKR (*p* < 0.05; OR 0.87) and living in an urban area (*p* < 0.05; OR 0.616) compared to a rural area were significant with practices. A positive attitude was observed regarding awareness and prevention of the disease. Awareness campaigns and providing health education could be key approaches to manage this disease in the general population of developing countries.

## 1. Introduction

Human taeniasis is the one of the leading foodborne parasitic disease, according to the World Health Organization (WHO) [1]. The causative agents are tapeworms from the Taeniidae family (subclass Eucestoda, order Cyclophyllidea) [2]. The adult tapeworms of the three responsible species are found in the small intestine of humans. Cattle serve as the vertebrate intermediate host of *Taenia saginata*, whereas pigs are the larval hosts for *T. asiatica* and *T. solium* [3]. The accidental entry of the eggs through contaminated food or water leads to the onset of cysticercosis, in which humans serve as an intermediate host for the parasite. In the human intestine, the worm takes between 5 and 12 weeks to reach adulthood [4]. *T. solium* can live for at least 25 years. Its eggs are spherical and within the shell are six-hooked tapeworm larvae. These larvae are small cysticerci, about 6–18 mm wide and 4–6 mm in length, and can be found in the muscle or subcutaneous tissues of their intermediate host (generally pigs). The cysticerci can also be found in other tissues, including those of the central nervous system, where they can grow much larger, sometimes many cm in diameter. Adult tapeworms have a median length of approximately 3 m; however, they can develop to up to 8 m in length. A single worm may harbor 800–1000 proglottids filled with eggs [5].

Neurocysticercosis (NCC), due to the development of cysts in the central nervous system (CNS), is frequently reported. NCC is considered the most common parasitic infection of the human nervous system. It is the most preventable cause of epilepsy in developing countries, and about 30% of cases are reported from underdeveloped countries [6]. Cysticercosis is prevalent in various countries and is associated with poverty and illiteracy, as well as the lack of competent diagnostic and management skills and effective preventative and control efforts. Cysticercosis imposes a significant economic burden owing to losses in the meat industry from porcine cysticercosis and hospitalization expenditure in managing NCC [7,8].

The prevalence of taeniasis and cysticercosis in developed countries is likely evolving, but a lack of solid data is one of the biggest barriers in determining the actual size of the problem. In one epidemiological investigation, females were found to have a greater prevalence (61%) than males (32%) [9]. The illness burden brought on by NCC is higher in areas where it is endemic. In Honduras, Ecuador, and Peru, recent controlled studies using computed tomography have revealed a strong association in the field between NCC and seizures, with nearly 30% of seizures attributable to NCC infection [10]. According to the WHO, taeniasis affects 500 million people worldwide, mostly in underdeveloped nations, and leads to the death of 50,000 people per year [11]. Asian countries, such as India, Pakistan, northern China, and Thailand, are affected by the disease [12]. In Nepal, *Taenia* cysts were discovered in pig flesh from Kangeswari, Kathmandu for the first time in 2019 [13].

The linked variables of a high frequency of infection include risk factors, production systems, food culture, insufficient regulatory mechanisms, and low priority in control programs. The increasing incidence of cysticercosis in pigs and humans is linked to a rapid rise in small-scale pig farming [9]. Cysticercosis is thought to be eradicable due to several factors, including the following: humans are the only definitive host and the only source of infection for intermediate hosts; domestic animals serve as primary intermediate hosts or reservoirs and are easily controlled; there are no significant wildlife reservoirs; and control interventions are readily available. There have been infrequent reports of cysticercosis, particularly the cerebral variant. Additionally, the racemose form has been documented.

Establishing effective control and elimination measures for many illnesses depends heavily on community knowledge, attitudes, and practices (KAPs) [14]. With the right information, people are more likely to adopt prevention measures, such as treating tapeworm infections and adopting better sanitation, cleanliness, and improved pig-rearing techniques, that may reduce the feco-oral spread of numerous infectious diseases. Data from a KAP survey can be used to identify knowledge gaps, cultural norms, or behavioral patterns that could be problematic for understanding and taking action while also hindering efforts to manage or eradicate the disease. However, there is little KAP information about *Taenia solium* cysticercosis in Asian countries [15,16], especially Pakistan. As a result, the current study sought to assess KAPs regarding taeniasis in Pakistan. The findings can serve as the foundation for the creation of a contextualized health education package that can be used locally to manage or eradicate taeniasis.

## 2. Materials and Methods

### 2.1. Study Site

The study was conducted among the rural and urban populations of the cities of Rawalpindi and Islamabad in Pakistan. Islamabad is located at the northern edge of the Potohar plateau at an elevation of 540 m, and it has a population of 2.015 million, while Rawalpindi lies on the Potohar plateau 9 miles southwest of Islamabad and has a population of approximately 2.908 million. The cities are known as the “twin cities” of Pakistan [17].

### 2.2. Study Design

The study aimed to explore the knowledge, attitudes, and practices (KAP) related to taeniasis among the general population through a descriptive cross-sectional approach. To obtain the requisite information, a pre-validated questionnaire that adhered to standardized protocols was devised. This questionnaire was formulated after a comprehensive evaluation of an English language version. Subsequently, the survey was rendered into informal language to guarantee clarity and understanding for the respondents. The study placed significant emphasis on inclusivity, whereby individuals from a wide range of ethnicities, religions, genders, and geographical locations were considered eligible to participate in the study. The assessment of KAP was restricted to individuals aged between 10 and 70 years, thereby ensuring a targeted sample size that included both adolescent and adult participants. The study focused on providing an extensive understanding regarding the knowledge, attitudes, and practices concerning taeniasis among a representative cross-section of the general public using these selection criteria.

### 2.3. Sample Size Calculation

We used the Raosoft calculator [18], assuming a 95% CI with a 5% margin of error and Z of 1.96, to calculate the sample size. The estimated population of Rawalpindi is 2.908 million, and that of Islamabad is around 2.015 million, and the required sample size was 770.

### 2.4. Data Collection

Data were collected to assess the KAPs related to taeniasis. A structured questionnaire was used, and responses were collected through both an online survey and door-to-door interviews. A wide range of participants, including those who lived in both urban and rural areas, were approached using the online survey.

### 2.5. Questionnaire

We used a self-designed KAP questionnaire as the survey tool to collect data from residents. This questionnaire was prepared in English and divided into four sections. The first section comprised questions gathering socio-demographic information including age, gender, area, residence, income, occupation, education, and number of family members. The rest of the questionnaire comprised three sections of questions to assess knowledge (n = 28), attitudes (n = 10), and practices (n = 7) regarding taeniasis.

### 2.6. Data Analysis

A Microsoft Excel file was used for sorting and storage purposes, and SPSS 24.0 was used for the statistical analysis. Descriptive statistics were used to calculate answer frequency and percentages. The Chi-square test was used to examine the association between two categorical variables. Non-parametric tests were used for non-categorical variables (Mann–Whitney U test and Kruskal–Wallis test). The level of significance was set at 0.05.

## 3. Results

The KAP questionnaire was circulated among 800 individuals in Rawalpindi and Islamabad, and 770 responses with complete information were received and included in the analysis.

### 3.1. Demographic Characteristics of Participants

**Figure 1:**
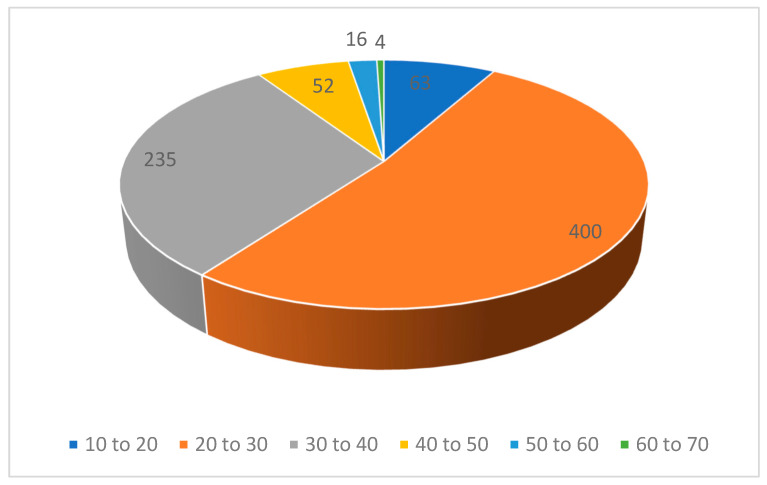
Age distribution of the participants (N = 770).

**Figure 2:**
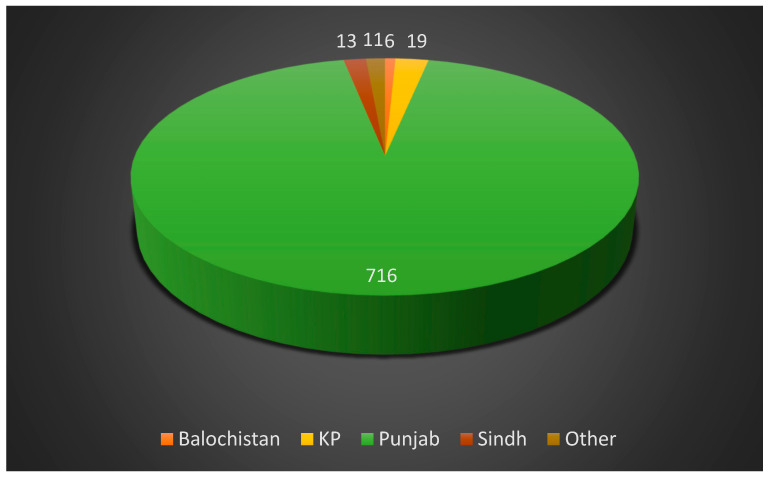
Distribution of participants according to province (N = 765).

The study population comprised more women (n = 547, 71%) than men (n = 220, 28.6%). In terms of age, 51.9% (n = 400) of participants were between 20–30 years old (Figure 1). In terms of religion, most respondents were Muslim (96.1%, n = 740). As far as the distribution of the participants is concerned, the majority were from Punjab (n = 716) (Figure 2). Owing to the distribution of the questionnaire among students, most participants were recorded as undergraduate students (59.61%). The respondents were concentrated in urban areas (86.4%), and 31.6% (n = 243) had a monthly household income of 31,000–50,000 PKR. The most frequent family size was 4–5 members (43.2%, n = 333; Table 1).

### 3.2. Knowledge of Participants about Taeniasis

A total of 28 questions assessed knowledge and its impact on taeniasis. The frequency and percentage of these participants showed that the largest percentage of people (44.4%) had little knowledge about the disease and its impact. In terms of diet, 49.6% (n = 382), of the participants cooked beef at home, and only a few of the respondents were non-vegetarian (6.4%, n = 49). Among the participants, 24.5% reported consuming uncooked meat, with the majority eating beef compared to pork (2.6%). Only 11.4% of respondents had seen proglottids in their feces. However, more respondents were aware of taeniasis as a diagnosis, and that tapeworm species causing taeniasis are normally found in meat (52.3%). They were also aware that transmission is linked to poor sanitation and consumption of infected beef and pork (Table 2).

### 3.3. Attitudes of Participants towards Taeniasis

Respondents reported specific attitudes toward the prevention of taeniasis. The inclination was toward avoiding eating raw, undercooked, or unhygienically prepared meat. Positive attitudes regarding awareness and prevention of disease were observed. Most respondents were willing to participate in campaigns (69.1%) and provide blood or feces samples (67.1%) as part of efforts to eliminate the disease. A large percentage of respondents understood that cattle and pigs should be vaccinated (66.5%), and that there should be proper disposal of animal waste other than open defecation. A need for community-level programs to ensure meat inspection was expressed by 61.3% of the participants, and 66.9% showed a willingness to check the internal temperature of food if awareness was raised and thermometers were introduced (Table 3).

### 3.4. Practices of Participants about Taeniasis

A significant proportion of the 770 respondents washed their hands before and after preparing food and washed meat properly before cooking. The majority kept raw meat separated from clean utensils (81.6%) and checked the internal temperature of the meat when it was cooking (75.1%). About 90% of the study population reported washing their hands after defecation. However, 70% ate food from stalls/vendors (Table 4).

### 3.5. Association between Knowledge and Socio-Demographic Characteristics of Participants

We used the independent variables of gender, age, province, residence, occupation, education, area, annual income, and knowledge as dependent variables. We applied binomial logistic regression to the independent variables with the dependent variables and obtained *p* values and odds ratios (ORs). In terms of the associations between knowledge and socio-demography, we observed a significant association between knowledge and having a family size of eight to nine members (*p* < 0.05; OR 0.782), as compared to two to three family members. Variables such as age, gender, province, residence, area, religion, and income were not significantly related to knowledge (i.e., *p* > 0.05). ORs and 95% confidence intervals are shown in Table 5.

### 3.6. Association between Attitudes and Socio-Demographic Characteristics of Participants

Using regression testing as part of our statistical analysis, we determined significant associations between knowledge and age, especially in the 20–30 years (*p* < 0.05; OR 0.574) and 30–40 years (*p* < 0.05; OR 0.553) age groups. We also found a significant relationship between knowledge and being a resident in Rawalpindi (*p* < 0.05; OR 0.68) and other cities (*p* < 0.05; OR 2.43), except Islamabad. At the same time, a significant association was seen between attitude and being neither employed nor a student (*p* < 0.05; OR 2.761). Statistical analysis of income showed that income ranges from 31,000–50,000 PKR (*p* < 0.05; OR: 0.574), 51,000–70,000 PKR (*p* < 0.05; OR 0.531), and above 70,000 PKR (*p* < 0.05; OR 0.42) were significantly related to attitude, as compared to income ranging from 10,000 to 30,000 PKR (Table 6).

### 3.7. Association between Practices and Socio-Demographic Characteristics of Participants

Statistical analysis of associations between practices and socio-demographic variables indicated that living in an urban area and income level were significant. Income of more than 70,000 Pkr (*p* < 0.05; OR 0.87) and living in an urban area (*p* < 0.05; OR 0.616), as compared to rural areas, were significantly related to practices (Table 7).

**Table 5:** Associations between knowledge and socio-demographic characteristics of participants.

Variables	Category	Knowledge		Estimate	SE	Z-Value	*p*-Value	Odds Ratio (95% CI)	*R*^2^*_mcf_*
		Good	Poor						
Age	10 to 20 (Base)	38	25	-	-	-	-	-	0.00247
	20 to 30	226	174	0.1527	0.277	0.569	0.570	1.170 (−0.385–0.6993)	
	30 to 40	140	95	0.0309	0.290	0.107	0.915	1.031 (−0.537–0.5989)	
	40 to 50	33	19	−0.1334	0.386	−0.345	0.730	0.875 (−0.891–0.6238)	
	50 to 60	11	5	−0.3697	0.598	−0.619	0.536	0.691 (−1.541–0.8017)	
	60 to 70	3	1	−0.6799	1.183	−0.575	0.565	0.507 (−2.999–1.6389)	
Gender	Female (Base)	325	222	-	-	-	-	-	0.00065
	Male	124	96	0.125	0.1614	0.776	0.438	1.133 (−0.191–0.442)	
	Not available	2	1	−0.312	1.2278	−0.254	0.799	0.732 (−2.719–2.095)	
Province	Balochistan (Base)	2	4	-	-	-	-	-	0.0111
	KP	5	14	0.336	1.011	0.333	0.739	1.400 (−1.64–2.317)	
	Punjab	424	292	−1.066	0.869	−1.226	0.220	0.344 (−2.77–0.638)	
	Sindh	9	4	−1.504	1.054	−1.427	0.154	0.222 (−3.57–0.562)	
	Other	7	4	−1.253	−3.35	−1.172	0.241	0.286 (−3.35–0.843)	
	Not available	4	1	−2.079	1.414	−1.470	0.141	0.125 (−4.85–0.692)	
Residence	Islamabad (Base)	135	85	-	-	-	-	-	0.00663
	Rawalpindi	283	222	0.220	0.165	1.3328	0.183	1.246 (−0.103–0.543)	
	Other	32	12	−0.518	0.366	−1.4169	0.157	0.596 (−1.235–0.199)	
	Not available	1	0	−13.103	535.411	−0.0245	0.980	0.0000020 (−1062.490–1036.283)	
Status	Employee (Base)	253	193	-	-	-	-	-	0.00351
	Student	152	95	−0.1993	0.1620	−1.2304	0.219	0.819 (−0.517–0.1182)	
	Other	44	31	−0.0795	0.2532	−0.3140	0.754	0.924 (−0.576–0.4168)	
	Not available	2	0	−13.2594	378.5929	−0.0351	0.972	0.0000068 (−0.755.324–728.7330)	
Religion	Christian (Base)	14	3	-	-	-	-	-	0.00653
	Hindu	3	1	0.442	1.318	0.335	0.738	1.556 (−2.1421–3.026)	
	Muslim	430	310	1.213	0.641	1.894	0.058	3.364 (−0.0422–2.469)	
	Other	1	3	1.639	1.318	2.002	0.055	14.00 (0.0551–5.223)	
	Not available	3	2	1.135	1.113	1.020	0.308	3.111 (−1.0459–3.316)	
Occupation	Business (Base)	93	53	-	-	-	-	-	0.00570
	Farmer	15	8	−0.0663	0.470	−0.141	0.888	0.936 (−0.9883–0.856)	
	Housewife	70	45	0.1205	0.257	0.468	0.639	1.128 (−0.3835–0.624)	
	Medical/paramedical Staff	19	12	0.1028	0.407	0.253	0.801	1.108 (−0.6948–0.900)	
	Teacher	88	68	0.3045	0.236	1.290	0.197	1.356 (−0.1580–0.767)	
	Other	161	132	0.3637	0.208	1.746	0.081	1.439 (−0.0446–0.772)	
	Not available	5	1	−1.0471	1.109	−0.944	0.345	0.351 (−3.2205–1.126)	
Education	Elementary (Base)	19	8	-	-	-	-	-	0.00529
	Secondary	25	17	0.479	0.526	0.912	0.362	1.583 (−1.5111–2.4032)	
	Higher Secondary	135	102	0.585	0.441	1.325	0.185	1.794 (−0.2805–1.4498)	
	Graduation	232	151	0.436	0.434	1.003	0.316	1.546 (0.4156–1.2866)	
	Not available	3	2	0.460	1.005	0.457	0.648	1.583 (−1.5111–2.4032)	
Area	Rural (Base)	48	47	-	-	-	-	-	0.00456
	Urban	395	270	−0.3594	0.220	−1.635	0.102	0.698 (−0.790–0.0715)	
	Not available	8	2	−1.3652	0.817	−1.672	0.095	0.255 (−0.790–0.0715)	
Income	10,000–30,000 (Base)	44	39	-	-	-	-	-	0.0106
	31,000–50,000	158	85	−0.4993	0.258	−1.937	0.053	0.607 (−1.005–0.00597)	
	51,000–70,000	126	84	−0.2848	0.261	−1.091	0.275	0.752 (−0.797–0.22705)	
	Above 70,000	100	96	0.0798	0.262	0.304	0.761	1.083 (−0.434–0.59384)	
	Not available	2	3	0.5261	0.939	0.560	0.575	1.692 (−1.314–2.36648)	
Family members	2 to 3 (Base)	54	24	-	-	-	-	-	0.00716
	4 to 5	195	138	0.465	0.269	1.727	0.084	1.592 (−0.0628–0.993)	
	6 to 7	151	110	0.494	0.275	1.794	0.073	1.639 (−0.0458–1.034)	
	8 to 9	30	31	0.844	0.335	2.379	0.017	2.325 (0.1486–1.539)	
	10 to 11	10	9	0.706	0.521	1.355	0.176	2.025	
	More than 11	10	5	0.118	0.600	0.196	0.844	1.125 (−1.0585–1.294)	
	Not available	1	2	1.504	1.249	1.204	0.229	4.500 (−0.9441–3.952)

**Table 6:** Associations between attitude and socio-demographic characteristics of participants.

Variables	Category	Knowledge		Estimate	SE	Z-Value	*p*-Value	Odds Ratio (95% CI)	*R*^2^*_McF_*
		Good	Poor						
Age	10 to 20 (Base)	32	31	-	-	-	-	-	0.00619
	20 to 30	257	143	−0.5545	0.273	−2.033	0.042	0.574 (−1.089–(−0.0199))	
	30 to 40	153	82	−0.5920	0.287	−2.064	0.039	0.553 (−1.154–(−0.0299))	
	40 to 50	29	23	−0.2001	0.376	−0.532	0.595	0.819 (−0.937–0.5371)	
	50 to 60	9	7	−0.2196	0.563	−0.390	0.697	0.803 (−1.324–0.8848)	
	60 to 70	3	1	−1.0669	1.182	−0.903	0.367	0.344 (−3.383–1.2496)	
Gender	Female (Base)	347	200	-	-	-	-	-	0.00601
	Male	136	84	0.0692	0.1647	0.4199	0.675	1.072 (−0.254–0.392)	
	Not available	0	3	15.1171	509.6521	0.0297	0.976	3.68 (−983.783–1014.017)	
Province	Balochistan (Base)	5	1	-	-	-	-	-	0.00533
	KP	13	6	0.836	1.20	0.696	0.486	2.308 (−1.519–3.191)	
	Punjab	452	264	1.072	1.10	0.976	0.329	2.920 (−1.081–3.224)	
	Sindh	6	7	1.764	1.23	1.435	0.151	5.833 (−0.644–4.172)	
	Other	5	6	1.792	1.25	1.432	0.152	6.000 (−0.661–4.245)	
	Not available	2	3	2.015	1.43	1.413	0.158	7.500 (−0.780–4.810)	
Residence	Islamabad (Base)	128	92	-	-	-	-	-	0.0203
	Rawalpindi	339	166	−0.384	0.166	−2.3077	0.021	0.681 (0.710-	
	Other	16	28	0.890	0.342	2.6027	0.009	2.435 (0.220–1.5600)	
	Not available	0	1	13.896	535.411	0.0260	0.979	1,080,000 (−1035.490–1063.2829)	
Status	Employee (Base)	300	146	-	-	-	-	-	0.0185
	Student	149	98	0.301	0.165	1.8297	0.067	1.351 (−0.0214–0.624)	
	Other	32	43	1.016	0.254	3.9932	<0.001	2.761 (0.5171–1.514)	
	Not available	2	0	−12.846	378.593	−0.0339	0.973	0.00000265 (754.8743–729.183)	
Religion	Christian (Base)	7	10	-	-	-	-	-	0.00920
	Hindu	1	3	0.7419	1.225	0.5910	0.555	2.100 (−1.719–3.2026)	
	Muslim	472	268	−0.9227	0.499	−1.8501	0.064	0.397 (−1.900–0.0548)	
	Not available	2	3	0.0488	1.037	0.0470	0.962	1.050 (−1.984–2.0820)	
	Other	1	3	0.7419	1.255	0.5910	0.555	2.100 (−1.719–3.2026)	
Occupation	Business (Base)	88	58	-	-	-	-	-	0.0179
	Farmer	10	13	0.6793	0.453	1.4983	0.134	1.972 (−0.209–1.5678)	
	Housewife	65	50	0.1545	0.253	0.6109	0.541	1.167 (−0.341–0.6503)	
	Medical/paramedical Staff	20	11	−0.1809	0.412	−0.4395	0.660	0.834 (−0.988–0.6260)	
	Other	203	90	−0.3965	0.211	−1.8766	0.061	0.673 (−0.811–0.0176)	
	Teacher	91	65	0.0804	0.234	0.3430	0.732	1.084 (−0.379–0.5400)	
	Not available	6	0	−14.1492	360.379	−0.0393	0.969	0.00000071 (−720.478–692.1797)	
Education	Elementary (Base)	13	14	-	-	-	-	-	0.0165
	Secondary	20	22	0.0212	0.494	0.0429	0.966	0.966 (−0.947–0.98897)	
	Higher Secondary	138	99	−0.4062	0.407	−0.9980	0.318	0.666 (−1.204–0.39158)	
	Graduation	254	129	−0.7516	0.400	−1.8788	0.060	0.472 (−1.536–0.03246)	
	Not available	5	0	−14.6402	394.775	−0.0371	0.970	0.00000043 (−788.385–759.110466)	
Area	Rural (Base)	57	38	-	-	-	-	-	5.6 × 10^−4^
	Urban	419	246	−0.127	0.224	−0.567	0.571	0.881 (−0.567–0.31255)	
	Not available	7	3	−0.442	0.721	−0.613	0.540	0.643 (−1.855–0.97158)	
Income	10–30 k (Base)	41	42	-	-	-	-		0.0173
	31–50 k	153	90	−0.5547	0.257	−2.162	0.031	0.574 (−1.058–(−1.058))	
	51–70 k	136	74	−0.6327	0.263	−2.407	0.016	0.531 (−1.148–(−0.1176))	
	Above 70 k	137	59	−0.8665	0.269	−3.219	0.001	0.420 (−1.394–(−0.3390))	
	Not available	2	3	0.3814	0.939	0.406	0.685	1.464 (−1.459–2.2216)	
Family members	2 to 3 (Base)	40	38	-	-	-	-	-	0.0121
	4 to 5	214	119	−0.5356	0.254	−2.1105	0.035	0.585 (−1.033–(−0.0382))	
	6 to 7	174	87	−0.6419	0.262	−2.4514	0.014	0.526 (−1.155–0.1287)	
	8 to 9	35	26	−0.2460	0.344	−0.7150	0.475	0.782 (−0.920–0.4283)	
	10 to 11	9	10	0.1567	0.512	0.3058	0.760	1.170 (−0.847–1.1607)	
	More than 11	8	7	−0.0822	0.565	−0.1456	0.884	0.921 (−1.190–1.0251)	
	Not available	3	0	−14.5148	509.652	−0.0285	0.977	0.00000049 (−1013.415–984.3851)

**Table 7:** Association between practices and socio-demographic characteristics of participants.

Variables	Category	Knowledge		Estimate	SE	Z-Value	*p*-Value	Odds Ratio (95% CI)	*R*^2^*_McF_*
		Good	Poor						
Age	10 to 20 (Base)	34	29	-	-	-	-	-	0.00399
	20 to 30	217	183	−0.1591	0.272	−0.0417	0.967	0.989 (−0.544–0.522)	
	30 to 40	137	98	−0.1759	0.285	−0.6167	0.537	0.839 (−0.735–0.383)	
	40 to 50	29	23	−0.0727	0.377	−0.1931	0.847	0.930 (−0.811–0.665)	
	50 to 60	12	4	−0.9395	0.630	−1.4907	0.136	0.391 (−2.175–0.296)	
	60 to 70	3	1	−0.9395	1.182	−0.7949	0.427	0.391 (−3.256–1.377)	
Gender	Female (Base)	303	244	-	-	-	-	-	0.00107
	Male	128	92	−0.114	0.1615	−0.704	0.481	0.893 (−0.430–0.2028)	
	Not available	1	2	0.910	3.3161	0.741	0.459	2.484 (−1.497–3.3161)	
Province	Balochistan (Base)	2	4	-	-	-	-	-	0.00593
	KP	6	13	0.0800	0.997	0.0803	0.936	1.083 (−1.87–2.034)	
	Punjab	407	309	−0.9686	0.869	−1.1142	0.265	0.380 (−2.67–0.735)	
	Sindh	8	5122	−1.1632	1.037	−1.1218	0.262	0.313 (−3.20–0.869)	
	Other	6	5	−0.8755	1.258	−0.8285	0.407	0.417 (−2.95–1.196)	
	Not available	3	2	−1.0986	1.258	−0.8731	0.383	0.333 (−3.56–1.368)	
Residence	Islamabad (Base)	122	98	-	-	-	-	-	0.00169
	Rawalpindi	286	219	−0.0479	0.163	−0.2942	0.769	0.953 (−0.367–0.2710)	
	Other	24	20	0.0367	0.332	0.1107	0.912	1.037 (−0.614–0.6870)	
	Not available	0	1	13.7851	535.411	0.0257	0.979		
Status	Employee (Base)	257	189	-	-	-	-	-	0.00450
	Student	137	110	0.0878	0.1599	0.5492	0.583	1.092 (−0.226–0.401)	
	Other	36	39	0.3874	0.2502	1.5482	0.122	1.473 (−0.103–0.878)	
	Not available	2	0	−13.2587	378.5929	−0.0350	0.972	0.0000175e (755.287–728.770)	
Religion	Christian (Base)	11	6	-	-	-	-	-	0.00829
	Hindu	1	3	1.705	1.261	1.3516	0.177	5.500 (−0.767–4.177)	
	Muslim	417	323	0.351	0.513	0.6838	0.494	1.420 (−0.655–1.356)	
	Other	0	4	15.172	441.372	0.0344	0.973	3,880,000 (−84.901–880.245)	
	Not available	3	2	0.201	1.044	0.1921	0.848	1.222 (−1.846–2.248)	
Occupation	Business (Base)	89	57	-	-	-	-	-	0.0119
	Farmer	15	8	−0.183	0.470	−0.3898	0.697	0.833 (−1.1033–0.737)	
	Housewife	63	52	0.254	0.253	1.0037	0.316	1.289 (−0.2417–0.79)	
	Medical/paramedical Staff	20	11	−0.152	0.412	−0.3696	0.712	0.859 (−0.9596–0.655)	
	Teacher	88	68	0.189	0.234	0.8017	0.423	1.207 (−0.2713–0.647)	0.00597
	Other	151	142	0.384	0.206	1.8645	0.062	1.468 (−0.0197–0.788)	
	Not available	6	0	−14.120	360.37	−0.0392	0.969	0.000073 (−720.4494–692.208)	
Education	Elementary (Base)	17	10	-	-	-	-	-	0.00701
	Secondary	19	23	0.722	0.505	1.429	0.153	2.058 (−0.268–1.711)	
	Higher Secondary	126	111	0.404	0.419	0.963	0.335	1.498 (−0.418–1.226)	
	Graduation	226	157	0.166	0.412	0.404	0.686	1.181 (−0.641–0.974)	
	Other	40	36	0.425	0.460	0.924	0.355	1.530 (−0.476–1.327)	
	Not available	4	1	−0.856	1.187	−0.721	0.471	0.425 (−3.182–1.471)	
Area	Rural (Base)	43	52	-	-	-	-	-	0.00538
	Urban	381	284	−0.484	0.221	−2.194	0.028	0.616 (−0.916–(−0.0516))	
	Not available	8	2	−1.576	0.817	−1.929	0.054	0.207 (−3.178–0.0249)	
Income	10–30 k (Base)	42	47	-	-	-	-	-	0.00538
	31–50 k	146	97	−0.3848	0.256	−1.505	0.132	0.681 (−0.886–0.116)	
	51–70 k	118	92	−0.2248	0.260	−0.865	0.387	0.799 (−0.734–0.285)	
	Above 70 k	106	90	−0.1395	0.262	−0.532	−0.595	0.870 (−0.653–0.374)	
	Not available	1	4	1.4104	1.139	1.238	0.216	4.098 (−0.823–3.644)	
Family members	2 to 3 (Base)	47	31	-	-	-	-	-	0.00644
	4 to 5	189	144	0.144	0.256	0.5624	0.574	1.155 (−0.358–0.6469)	
	6 to 7	147	114	0.162	0.263	0.6160	0.538	1.176 (−0.353–0.6772)	
	8 to 9	30	31	0.449	0.345	1.3008	0.193	1.567 (−0.228–1.1254)	
	10 to 11	8	11	0.735	0.519	1.4152	0.157	2.085 (−0.283–1.7520)	
	More than 11	8	7	0.283	0.567	0.4985	0.618	1.327 (−0.829–1.3938)	
	Not available	3	0	−14.150	509.652	−0.0278	0.978	0.00000071 (−1013.050–984.7500)	

## 4. Discussion

Taeniasis is widespread in East, Southeast, and South Asia across the region’s rich diversity of cultural, traditional, and behavioral norms [19,20]. Many studies have discussed the prevalence of soil-transmitted helminths and other underdiagnosed tropical diseases [21,22,23], but the three co-occurring human Taenia species have rarely been investigated in depth. It is unclear how widespread the problem is in East, Southeast, and South Asia, and incidence rates reported by the various countries and territories vary considerably [5,24,25]. The significant findings on KAPs relating to taeniasis in these countries, however, point to issues with sanitation at an individual, household, and community level. Cysticercosis can be prevented and controlled through better sanitation and health education, the application of food safety precautions, and the use of improved and standardized diagnostic tests, as well as through the reporting of infections at the species level [26]. The holistic approach known as “One Health” can be used to apply these methods, and this approach considers the well-being of humans, animals, and the planet. Most intestinal infections are asymptomatic. Symptoms are often modest and may include stomach discomfort, anorexia, weight loss, or malaise. Cysticercosis has a widespread impact on several essential organs (e.g., brain, eye, heart); however, it has a low death rate, and death is usually caused by complications such as encephalitis, increased intracranial pressure due to edema and/or hydrocephalus, or stroke. The infection affects people of all ages, sexes, and races equally [27].

### 4.1. Socio-Demographic Characteristics

In our study assessing KAPs regarding taeniasis, which is the first of its kind in Pakistan, we sought to describe the socio-demographic factors of the study population, including gender, education, residency marital status, age, and income. As the questionnaire was circulated among students, most of the participants were unemployed and between 20–30 years of age. The major concentration of respondents was in urban areas, and most respondents had a monthly household income of 31,000–50,000 PKR. A similar cross-sectional study was conducted in Punjab, India, comprising a survey questionnaire related to zoonotic diseases that was distributed to 859 participants. The majority were male farmers [28]. In another study, a structured questionnaire was circulated to collect socio-demographic variables and information on knowledge and attitudes regarding taeniasis/cysticercosis, raw meat consumption, latrine usage, and taeniasis treatment practices in two small towns in Ethiopia. The majority of the 195 participants were also male [29]. Food safety KAPs among 772 elementary schoolchildren were surveyed in southern Taiwan, with mostly female respondents [30]. In another cross-sectional study that was conducted in Ibadan, Nigeria, most of the participants were male [31]. In a KAP analysis relating to taeniasis disease that was conducted in South Africa, most participants were male and had only primary school education, with some having obtained secondary education [32]. A similar cross-sectional study conducted in Tanzania related to taeniasis also had mostly male respondents [33].

### 4.2. Knowledge

Our study assessed the basic knowledge of participants about the cause of the disease and the parasite’s intermediate host. One study related to taeniasis that was conducted in Tanzania demonstrated knowledge about cysticercosis, particularly among cattle and pig keepers. Many participants had heard about tapeworm (*T. solium* taeniasis), and their knowledge of the signs and symptoms of the disease was good. Although most of the participants knew about epilepsy, none knew about the relationship or link between cysticercosis and epileptic seizures [34]. In another study conducted on farmer awareness and practices regarding taeniasis with 294 participants, only a small number knew about taeniasis disease [35]. The cross-sectional study conducted in small towns in Ethiopia demonstrated that meat industry workers and a large number of community members in both study areas had heard of human taeniasis [36]. Respondents purchasing pork from home slaughter were about four times less likely to demonstrate good knowledge in a study conducted in Nigeria [37]. In the KAP analysis conducted in South Africa, half of the respondents indicated no knowledge of cysticercosis in pigs, and the majority had never heard of NCC [37]. In a KAP study in Tanzania, the average number of respondents had heard of the pork tapeworm (*T. solium* taeniasis), and many (n = 163, 65%) were familiar with the signs and symptoms of the infection. However, only a few participants had accurate knowledge of the mode of transmission. Only a small number of respondents reported transmission through improperly cooked pork, and many participants falsely cited contaminated water [38].

### 4.3. Attitudes

In terms of attitudes toward the prevention of taeniasis disease, the inclination of many respondents was towards avoiding eating raw, undercooked, or unhygienically prepared meat. Positive attitudes towards awareness and prevention of disease in the community were observed. Most of the respondents were willing to participate in campaigns and provide blood and feces samples as part of efforts to eliminate it. Our results on respondents’ attitudes to taeniasis disease treatment, prevention, control, and the advantage of vaccination were consistent with a similar study conducted in Ethiopia [31]. A study performed in India found that the attitude of respondents towards disease control possibilities was better in those educated at college and university level when compared to illiterate people (*p* < 0.05) [39]; however, illiterate people were not included in our study. In contrast, the attitude towards the low-risk perception of cysticercosis is indicative of a positive trend in the Tanzanian study on taeniasis [16]. In the Taiwanese KAP analysis related to food safety, the attitude among students was not quite positive [15]. However, in a cross-sectional study conducted on smallholder farms in South Africa, results on the attitudes of individuals were not encouraging, and the community appeared to need more awareness [37]. Communities that are in underdeveloped countries with low literacy rates or are located in peripheral areas need improved understanding and greater awareness of taeniasis through awareness campaigns.

### 4.4. Practices

Most of the participants in our study were practicing hygiene by washing their hands before and after cooking food. The same study was conducted in Swat, Pakistan, and most of the surveyed population kept raw meat separated from clean utensils and checked the internal temperature of the meat. Public education to improve hygiene practices, curb risky culinary habits, promote taeniasis treatment, and discourage backyard slaughtering were suggested in a study conducted in small towns in Ethiopia [38]. Only hand washing before eating was significantly promoted in the practice domain (*p* < 0.001) in the study that was carried out relating to food safety in southern Taiwan [39,40], and poor practices were observed related to taeniasis in the KAP survey that was conducted in Nigeria. The majority of farmers in South Africa practiced a free-ranging system, as reported in some taeniasis studies, while a small number practiced a semi-intensive system [40,41].

## 5. Conclusions

KAPs have an enormous impact on the control of communicable diseases and in informing suitable policies. Owing to a lack of focus on awareness campaigns among the general population, many people are unaware of taeniasis. Our results demonstrated the importance of awareness of handling animals, keeping animals as pets, consuming raw meat, and handling infected animals. Respondents with lower educational levels and those who owned livestock had higher contact with animals but tended to consume more raw meat, not have pre-exposure vaccinations, and not take immediate action with infected animals. This study offers important new information about KAPs associated with taeniasis in the general population. The results show the need for focused education and awareness campaigns, especially among particular age groups and geographic areas. Taeniasis prevention initiatives should be tailored to the various professional and educational backgrounds of those involved. The study’s findings advance knowledge of taeniasis’ KAPs and can direct public health initiatives aimed at lessening the burden of this parasitic infection. In conclusion, populations with lower educational standing should be offered awareness and training programs on the transmission, treatment, prevention, and management of taeniasis, and related information. Additionally, strengthening intersectoral collaboration for the prevention and control of common zoonotic diseases is important.

While the focus of our research was to provide insights into the current state of knowledge and practice, we recognize the importance of conducting extensive research to produce significant results. Understanding the pathogenesis of taeniasis, identifying novel diagnostic techniques, and comparing the efficacy of various treatment options are all possible outcomes of intensive research. These areas of intensive research contribute to the advancement of disease knowledge, the improvement of diagnostic accuracy, and the development of more targeted and effective treatments.

## 6. Limitations

The research has utilized self-reported information, which is susceptible to both recall bias and social desirability bias. The reliability of data on participants’ knowledge, attitudes, and practices related to taeniasis may have been compromised by memory and reporting biases, which could have introduced some degree of error. The responses provided by the participants were influenced by social desirability bias, resulting in an overestimation of their knowledge, positive attitudes, or healthy practices about taeniasis. The presence of bias has the potential to compromise the precision and validity of the results. As the research utilized cross-sectional data gathering, it did not account for the evolution of knowledge, attitudes, and practices over a period. Adopting a longitudinal approach would yield a more all-encompassing comprehension of the aforementioned factors.

## Figures and Tables

**Table 1:** Socio-demographic characteristics of participants.

Variables	Scale	No (N)	Frequency (%)
Age	10 to 20	63	8.2
	20 to 30	400	51.9
	30 to 40	235	30.5
	40 to 50	52	6.8
	50 to 60	16	2.1
	60 to 70	4	0.5
Gender	Female	547	71.0
	Male	220	28.6
	Not available	3	0.4
Province	Balochistan	6	0.7
	KP	19	2.46
	Punjab	716	92.9
	Sindh	13	1.6
	Other	11	1.4
	Not available	5	0.6
Residence	Islamabad	220	28.6
	Rawalpindi	505	65.6
	Other	44	5.7
	Not available	1	0.1
Status	Employee	446	57.9
	Student	247	32.1
	Other	75	9.7
	Not available	2	0.3
Religion	Christian	17	2.2
	Hindu	4	0.5
	Muslim	740	96.1
	Other	4	0.5
	Not available	5	0.6
Occupation	Business	146	18.9
	Farmer	23	3.0
	Housewife	115	14.9
	Medical/paramedical staff	31	4.0
	Teacher	156	20.3
	Other	293	38.1
	Not available	6	0.8
Education	Elementary	27	3.5
	Secondary	42	5.5
	Higher secondary	237	30.8
	Graduation	459	59.61
	Not available	5	0.6
Area	Rural	95	12.3
	Urban	665	86.4
	Not available	10	1.3
Income	10–30 k	83	10.8
	31–50 k	243	31.6
	51–70 k	210	27.3
	Above 70 k	196	25.5
	Not available	38	5.0
Family Members	2–3	78	10.1
	4–5	333	43.2
	6–7	261	33.9
	8–9	61	7.9
	10–11	19	2.5
	More than 11	15	1.9
	Not available	3	0.4

**Table 2:** Knowledge of participants about taeniasis.

Variables	Scale	No (N)	Frequency (%)
Are you non-vegetarian?	Yes	49	6.4
	No	715	92.8
	Not available	6	0.8
Type of current food availabile	Home	595	77.3
	Restaurants	57	7.4
	Fast food	54	7.0
	Vendors/stalls	63	8.2
	Not available	1	0.1
Do you choose to eat food outside more?	Yes	282	36.6
	No	485	63.0
	Not available	3	0.4
Have you ever eaten uncooked meat?	Yes	189	24.5
	No	241	31.3
	Maybe	338	43.9
	Not available	2	0.2
Do you own livestock?	Yes	123	16.0
	No	632	82.1
	Not available	15	1.9
Do you cook beef at home?	Yes	382	49.6
	No	383	49.7
	Not available	5	0.6
Do you eat pork?	Yes	20	2.6
	No	727	94.4
	Not available	23	3.0
Do you know about zoonotic disease?	Yes	198	25.7
	No	263	34.2
	Maybe	306	39.7
	Not available	3	0.4
Have you ever been infected with taeniasis disease?	Yes	146	19.0
	No	618	80.3
	Maybe	6	0.8
Have any of your family members been diagnosed with this disease?	Yes	141	18.3
	No	300	39.0
	Not sure	329	42.7
Do you know eating undercooked food can cause disease in humans?	Yes	240	31.2
	No	212	27.5
	Maybe	317	41.2
	Not available	1	0.1
Do you know about taeniasis disease?	Yes	170	22.1
	No	254	33.0
	Maybe	342	44.4
	Not available	4	0.5
If yes, do you know about the symptoms?	Yes	194	25.2
	No	569	73.9
	Not available	7	0.9
Do you know this disease is caused by eating raw/undercooked food?	Yes	263	34.2
	No	499	64.8
	Not available	8	1.0
Taeniasis is a ____________ infection?	Bacterial	83	10.8
	Parasitic	369	47.9
	Viral	178	23.1
	Other	139	18.1
	Not available	1	0.1
Have you ever seen noodle-like proglottids in feces?	Yes	88	11.4
	No	588	76.4
	Not available	94	12.2
Tapeworm species causing taeniasis are normally found where?	Eggs	178	23.1
	Meat	403	52.3
	Vegetables	26	3.4
	Not sure	162	21.0
	Not available	1	0.1
Transmission of tapeworm species of taeniasis is linked with what?	Consumption of infected beef and pork	213	27.7
	Poor sanitation	106	13.8
	Both	449	58.3
	Not available	2	0.3
The intermediate hosts of taeniasis are what?	Both	445	57.8
	Cattle	132	17.1
	Pig	190	24.7
	Not available	3	0.4
How can taeniasis be diagnosed?	Direct microscopy of expelled eggs in feces	380	49.4
	Blood test	177	23.0
	Not sure	210	27.3
	Not available	3	0.4
Humans can become infected with species causing taeniasis by what?	Eating raw/undercooked meat	478	62.4
	Eating raw/undercooked vegetables	42	5.5
	Poor sanitation	132	17.2
	Not sure	114	14.9
Infection from tapeworm species of taeniasis may cause what?	Abdominal pain	95	12.3
	Loss of appetite	71	9.2
	Loss of weight	45	5.8
	Upset stomach	69	9.0
	All of above	446	57.9
	None of above	44	5.7
If one person has taeniasis, can this be passed on to other people in the family?	Yes	258	33.5
	No	126	16.3
	Not sure	380	49.3
	Not available	6	0.8
Meat hygiene can be achieved through what?	Correct cooking	145	18.8
	Proper inspection of meat	174	22.6
	All of above	314	40.8
	Not sure	133	17.3
	Not available	4	0.5
Which of the following is the effective treatment of the disease?	Drugs	164	21.3
	Surgery	111	14.4
	Depends on the severity of the infection	304	39.5
	Not sure	188	24.4
	Not available	3	0.4
How long does taeniasis last?	Less than 1 year	403	52.3
	2–3 years	78	10.1
	3–4 years	47	6.1
	Not available	139	31.0
	Not sure	3	0.4
Do you know that people with this disease may remain asymptomatic for many years?	Yes	479	62.2
	No	283	36.8
	Not available	8	1.0

**Table 3:** The attitude of participants towards taeniasis.

Variables	Scale	No (N)	Frequency (%)
Do you think you might become infected with this disease by eating unhygienic, raw, or undercooked meat?	Yes	434	56.4
	No	106	13.8
	Maybe	229	29.7
	Not available	1	0.1
Do you think there should be campaigns and programs on awareness and control of this disease?	Yes	587	76.2
	No	83	10.8
	Not sure	99	12.9
	Not available	1	0.1
Is there a need for proper treatment facilities for this disease?	Yes	550	71.4
	No	78	10.1
	Maybe	139	18.1
	Not available	3	0.4
Do you think cattle and pigs (the intermediate hosts of this disease) should be vaccinated?	Yes	512	66.5
	No	85	11.0
	Maybe	171	22.2
	Not available	2	0.3
If there was a mass screening program for taeniasis that involved providing stool and blood samples, would you participate?	Yes	515	67.1
	No	251	32.6
	Not available	2	0.3
If there were a community-based intervention program to eliminate taeniasis, would you participate?	Yes	535	69.5
	No	88	11.4
	Maybe	142	18.4
	Not available	5	0.6
If you were asked to use a food thermometer to measure the internal temperature of cooked food, would you do so?	Yes	515	66.9
	No	91	11.8
	Maybe	161	20.9
	Not available	3	0.4
At the community level, what can be done to prevent transmission of disease?	Ensuring meat inspection	472	61.3
	Banning the use of all meats	174	22.6
	Banning cultivation of vegetables	36	4.7
	Not sure	87	11.3
	Not available	1	0.1
If you were asked to participate in providing a feces sample to aid in disease prevention, would you participate?	Yes	539	70.0
	No	108	14.0
	Maybe	123	16.0
Do you think there should be proper disposal of animal waste other than open defecation?	Yes	589	76.5
	No	58	7.5
	Maybe	118	15.3
	Not available	5	0.6

**Table 4:** Practices of participants regarding taeniasis.

Variables	Scale	No (N)	Frequency (%)
Do you wash your hands before and after preparing food?	Yes	227	29.5
	No	401	52.1
	Maybe	142	18.4
Do you wash meat properly before cooking it?	Yes	700	90.9
	No	69	9.0
	Not available	1	0.1
Do you keep raw meat separated from clean utensils or ready-to-eat food?	Yes	628	81.6
	No	70	9.1
	Maybe	71	9.2
	Not available	1	0.1
Do you eat food from stalls/vendors or at restaurants?	Yes	539	70.0
	No	230	29.9
	Not available	1	0.1
Do you wash your hands with soap after defecation?	Yes	687	89.2
	No	80	10.4
	Not available	3	0.4
Do you check the internal temperature of the meat when cooking to ensure it is completely cooked?	Yes	578	75.1
	No	183	23.8
	Not available	9	1.2

## Data Availability

The data that support the findings of this study are openly available on request.
